# Embedded Wireless Flexible Sensor for Monitoring Interface Stress of Solid Rocket Motor

**DOI:** 10.3390/s26092807

**Published:** 2026-04-30

**Authors:** Bei Yan, Xiaozhou Lü, Kecai Ding, Yipeng Heng, Yong Li

**Affiliations:** 1 School of Aerospace Science and Technology, Xidian University, Xi’an 710071, China; yanbei@xidian.edu.cn (B.Y.); 23131213874@stu.xidian.edu.cn (K.D.); 25131213448@stu.xidian.edu.cn (Y.H.); 2 State Key Laboratory for Strength and Vibration of Mechanical Structures, Shaanxi Engineering Research Centre of NDT and Structural Integrity Evaluation, School of Aerospace Engineering, Xi’an Jiaotong University, Xi’an 710049, China; yong.li@mail.xjtu.edu.cn

**Keywords:** solid rocket motor, interface stress, health monitoring, embedded wireless flexible sensor

## Abstract

Solid rocket motor (SRM) is a reliable and cost-effective aerospace propulsion system, by virtue of its advantages in terms of simple structure, long storage life, low cost, and ease of manufacturing. However, cracks and interfacial delamination may occur at the interface owing to the interface stress resulting from the complex service scenarios throughout the entire life cycle of the SRM. Therefore, it is crucial to monitor the interface stress for health assessment of the SRM. To achieve non-destructive in situ monitoring of interface stress, this paper proposes a novel embedded wireless flexible sensor (EWFS). Through theoretical analysis, the expression of the relationship between the input and output signals of EWFS is formulated. The response patterns of the output signals under different interface stresses are investigated. A prototype of the EWFS comprising the flexible printed circuit board (FPCB) and polydimethylsiloxane (PDMS) is fabricated, along with an interface stress-testing system established for experiments. The experimental results indicate that the EWFS exhibits a sensitivity of 27.2 mV·MPa^−1^, a linearity error of 1.73%, a maximum hysteresis error of 2.67%, and a stability error of 0.023%.

## 1. Introduction

Solid rocket motor (SRM) is regarded as a reliable and cost-effective propulsion system in the aerospace field due to simple structure, long storage life, relatively low cost, and ease of manufacturing [1,2,3]. Throughout the entire life cycle of solid rocket motors, including production, storage, transportation, and ignition launch, the propellant grain is subjected to complex service environments such as long-term static gravity, transportation vibrations, and changes in ambient temperature [4,5,6]. Since the coefficient of thermal expansion and the modulus of the case material are orders of magnitude larger than those of the propellant, the propellant grain is subjected to significant radial stress [7]. The stress may cause internal cracks and propellant–liner interface debonding, affecting the reliability and integrity of the SRM [8,9,10]. Therefore, to conduct health monitoring of SRM grain throughout the entire lifecycle to evaluate the health status is of significant importance [11,12].

Currently, the health monitoring of SRM propellant mainly includes modeling analysis, sampling testing, and embedded sensor monitoring. The modeling analysis is a method that utilizes key parameters to establish a simulation model of SRM grain. By setting external load parameters, it simulates the service environment of SRM to obtain the stress state of the grain. Zhang et al. developed a creep damage constitutive model based on the generalized Kelvin model and continuum damage mechanics theory and established a three-dimensional SRM model containing the case, the insulator, and the propellant grain for calculating the mechanical response of the grain under thermal and gravitational loads and analyzing the stress concentration region in the grain [13]. Zhou et al. proposed a stochastic constitutive model with a lognormally distributed random parameter Lambda to analyze the structural integrity of an HTPB propellant grain. The results indicated that the stress–strain curves predicted by the stochastic constitutive model have a good agreement with the experimental curves [14]. Lei et al. calibrated the finite element model using tensile tests of solid propellants, which enabled an accurate prediction of the stress–strain response of interface debonding, verifying that interface debonding is induced by propellant breakage [15]. Although the modeling analysis gives the mechanical properties of the propellant grain at a low cost, the simulation model is unable to accurately simulate the complex force and thermal loads throughout the entire life cycle of SRM, resulting in limited engineering application value.

Non-destructive testing techniques are used to evaluate defects such as the propellant aging, cracks, and delamination without causing damage to the structural integrity of SRM. Chai et al. investigated deformation and fracture of solid propellant under quasi-static tensile loading by in situ synchrotron X-ray micro computed tomography and CT-image-based finite element method modeling [16]. Chu et al. proposed a nonlinear ultrasonic method to investigate and monitor in real time the nonlinear dynamic response of solid propellant. The dewetting damage has evolved by the nonlinear ultrasonic parameter such as the frequency peak and propagation distance [17]. Yan et al. proposed a multi-scale image enhancement algorithm based on low-passing filtering and nonlinear transformation for infrared testing image of the de-bonding defect in solid propellant rocket motors [18]. However, non-destructive testing techniques also have limitations in terms of high costs and real-time self-sensing.

In order to overcome the above shortcomings, recently, the application of embedded sensors in solid rocket engines has been studied. Shi et al. introduced a novel flexible three-dimensional stress sensor which was developed by integrating multiple pressure-sensing elements and encapsulating the force-concentrating layers for detecting multiaxial stress differences, the sensor exhibited a sensitivity coefficient of 1.5%/kPa at a pressure of 300 kPa, a maximum hysteresis error of 3.98%, and a stability error of 0.17% [19]. Nevertheless, the sensor is bulky in dimension (22 mm × 22 mm × 22 mm) and relies on wired connection. Duan et al. introduced a wired capacitive flexible sensor for measuring interface stress and investigated the effects of flexible interlayer modulus and electrode plate modulus on the interfacial stress field through simulation. The results demonstrated that a flexible interlayer modulus ranging from 5 MPa to 8 MPa delivered optimal sensor performance, while an electrode plate modulus of approximately 3 GPa was identified to maintain the favorable linearity of capacitance measurements. By comparing rigid sensors with the proposed flexible sensor, it is found that the flexible structure can significantly improve the internal stress state [20]. Su et al. presented a multiplexed wired interface stress sensor that integrates a pre-stretched fiber Bragg grating with a clamped–clamped beam. The sensor showed a validated embedded linear range of ±0.5 MPa under SRM-representative interface conditions [21]. The dual bond stress and temperature (DBST) sensor is employed for monitoring radial stress and temperature at the insulation/propellant interface due to its advantages of long-term stability, low power, and high precision [22,23]. Le et al. employed a finite element model to investigate the effect of delaminations on the radial stress distribution along the bondline during the cooling process of a solid rocket motor composed of propellant, insulation, and casing. A relationship is established between the debond angle, the number of DBST sensors, and the required sensor accuracy. A quantitative mapping is obtained between the debond size and the sensor data to inversely estimate the extent of the delamination [24].

However, the rigid sensors such as DBST and fiber Bragg grating have a modulus much higher than that of SRM components. This significantly alters the local stress field and interface strength. At present, flexible interface stress sensors adopt wired connection methods for power supply and stress signal transmission. The wiring of flexible interface stress sensors at the interface and lead-through holes in the case will damage the integrity and reliability of SRM.

In an effort to address the above issues, in light of the fact that aramid-fiber cases with ultra-low conductivity have been widely applied in SRMs [25,26,27], this paper proposes an embedded wireless flexible sensor (EWFS) based on the electromagnetic induction principle. The components of the EWFS on the inner and outer sides of the aramid-fiber case are connected wirelessly for non-destructive monitoring of the case–insulator interface stress in the SRM with aramid-fiber case. The feasibility and applicability of EWFS are investigated through numerical simulations and experiments. The rest of the paper is organized as follows: Section 2 introduces the composition of EWFS and elaborates the basic principles of EWFS for interface stress monitoring. In Section 3, the expression for the output signal of EWFS is derived through theoretical analysis, and the correlation between interfacial stress and the signal is analyzed. In Section 4, an EWFS system for interface stress monitoring is established, and the sensitivity, linearity, hysteresis error, repeatability, and stability of the EWFS are analyzed.

## 2. The Basic Principle of EWFS

**Figure 1:**
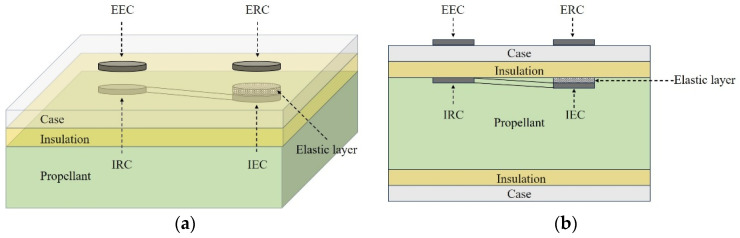
The schematic illustration of EWFS: (**a**) axonometric drawing; (**b**) section view.

The schematic illustration of the proposed EWFS is shown in Figure 1. The EWFS consists of an external excitation coil (EEC), an internal receiving coil (IRC), an internal excitation coil (IEC), an elastic layer and an external receiving coil (ERC). The sensor structure is a split type, with the EEC and the ERC located on the outer surface of the case, and the IRC, the elastic layer and the IEC located on the inner side of the thermal insulation layer. The elastic layer is situated between the IEC and the thermal insulation layer. It is noteworthy that the EEC and the IRC are coaxially distributed. The IEC, the elastic layer and the ERC are also positioned coaxially.

According to the law of electromagnetic induction, an alternating voltage source drives the EEC, thereby generating a varying magnetic field around the EEC. The varying magnetic field induces a changing magnetic flux in the IRC, resulting in an alternating electromotive force (EMF) in the IRC. Since the IRC and the IEC form a closed circuit, an alternating current is generated within the circuit, and a varying magnetic field is thus generated around the IEC. Consequently, an EMF is also induced in the ERC.

The thickness of the elastic layer varies with the interfacial stress, indicating a corresponding variation in the distance between the IEC and the ERC. This consequently brings about a change in the EMF in the ERC. Once the relationship between interfacial stress and the induced voltage has been established via calibration, the interfacial stress can be retrieved from the induced voltage.

## 3. Analytical Expressions and Simulations of EWFS

### 3.1. Analytical Expressions of EWFS

**Figure 2:**
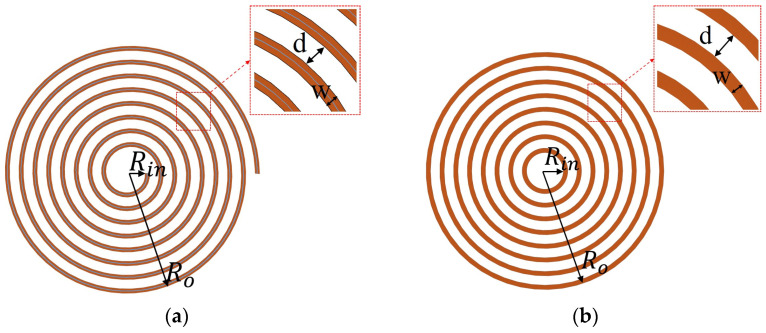
The planar coil: (**a**) Archimedean planar spiral coil; (**b**) concentric circular coil.

This paper employs the flexible printed circuit board (FPCB) to fabricate the EEC, the IRC, the IEC and the ERC, in order to ensure that the coils in the system are as thin as possible. The planar spiral coil (PSC) in the FPCB features an Archimedean planar spiral layout as shown in Figure 2a, where a single continuous conductor is wound around a central axis with uniform spacing between adjacent turns.

For an Archimedean planar spiral coil with *N* turns, where the inner edge radius of the innermost conductor is *R_in_*, conductor width is *w*, and turn spacing is *d*, it can be equivalently represented as *N* of concentric circular coil [28,29], as shown in Figure 2b. The equivalent radius *R*_1_ of the first turn is typically taken as the radius of its conductor centerline that is written as

(1) $$R_{1}=R_{in}+\frac{w}{2}$$

Since the radial spacing of the Archimedean spiral is uniform, the radius of the wire centerline increases by a fixed amount of *w* + *d* with each additional turn. Therefore, the equivalent radius *R_k_* of the *k*-th circular loop is written as

(2) $$R_{k}=R_{in}+(k-1)(w+d)+\frac{w}{2}$$

The outer radius *R*_0_ of the coil is expressed as

(3) $$R_{o}=R_{in}+(N-1)d+N\cdot w$$

**Figure 3:**
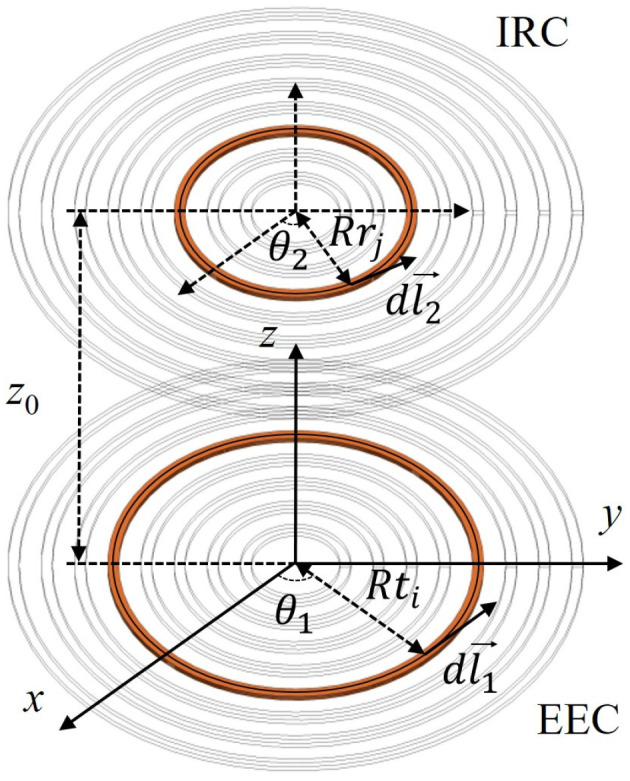
Equivalent model of the EEC and the IRC.

The mutual inductance between the EEC and the IRC can be obtained by taking the sum of the mutual inductances between each equivalent circular ring of the EEC and each equivalent circular ring of the IRC, the equivalent model of the EEC and the IRC is shown in Figure 3. It is noteworthy that the parameters of the EEC, the IRC, the IEC and the ERC are consistent in this paper. The Neumann equation provides an integral form based on the interaction between current elements to calculate the mutual inductance between any two coaxial circular loops, with its expression formulated as [30,31].

(4) $$M=\frac{\mu_{0}}{4\pi }\oint_{c_{1}}\oint_{c_{2}}\frac{1}{\left|\vec{r_{1}}-\vec{r_{2}}\right|}d\vec{l_{1}}d\vec{l_{2}}$$

where *μ*_0_ is the permeability of vacuum, *c*_1_ is the *i*-th turn equivalent circular coil of the EEC, *c*_2_ is the *j*-th turn equivalent circular coil of the IRC, $\vec{r_{1}}$ represents the position of line element $d\vec{l_{1}}$, and $\vec{r_{2}}$ represents the position of line element $d\vec{l_{2}}$.

As shown in Figure 3, the expressions for $\vec{r_{1}}$, $\vec{r_{2}}$, and the distance between them are individually written as

(5) $$\begin{array}{l} \vec{r_{1}}=(Rt_{i}\cos\theta_{1},Rt_{i}\sin\theta_{1},0)\\ \vec{r_{2}}=(Rr_{j}\cos\theta_{2},Rr_{j}\sin\theta_{2},z_{0})\\ \left|\vec{r_{1}}-\vec{r_{2}}\right|=\sqrt{Rt_{i}^{2}+Rr_{j}^{2}+z_{0}^{2}-2Rt_{i}Rr_{j}\cos(\theta_{1}-\theta_{2})} \end{array}$$

where *Rt_i_* is the radius of *i*-th turn equivalent circular coil of the EEC, *Rr_j_* is the radius of *j*-th turn equivalent circular coil of the IRC, and *z*_0_ is the sum of the case thickness and the insulation thickness. The expressions for *Rt_i_*, *Rr_j_*, $d\vec{l_{1}}$ and $d\vec{l_{2}}$ are formulated as

(6) $$\begin{array}{l} Rt_{i}=R_{in}+(i-1)(w+d)+\frac{w}{2},(i=1,2,\cdots ,N)\\ Rr_{j}=R_{in}+(j-1)(w+d)+\frac{w}{2},(j=1,2,\cdots ,N)\\ d\vec{l_{1}}=(-Rt_{i}\sin\theta_{1},Rt_{i}\cos\theta_{1},0)d\theta_{1}\\ d\vec{l_{2}}=(-Rr_{i}\sin\theta_{2},Rr_{i}\cos\theta_{2},0)d\theta_{2} \end{array}$$

Therefore, we come across the mutual inductance between the *i*-th turn equivalent circular coil of EEC and the *j*-th turn equivalent circular coil of the IRC written as

(7) $$M(Rt_{i},Rr_{j},z_{0})=\frac{\mu_{0}}{4\pi }\int_{0}^{2\pi }\int_{0}^{2\pi }\frac{Rt_{i}Rr_{j}\cos(\theta_{1}-\theta_{2})}{\sqrt{Rt_{i}^{2}+Rr_{j}^{2}+z_{0}^{2}-2Rt_{i}Rr_{j}\cos(\theta_{1}-\theta_{2})}}d\theta_{1}d\theta_{2}$$

By integrating Equation (7), the mutual inductance between EEC and IRC is thus expressed as

(8) $$M_{1}=\sum_{i=1}^{N}\sum_{j=1}^{N}M(Rt_{i},Rr_{j},z_{0})$$

Harold Wheeler proposed an empirical formula for calculating the self-inductance of hollow coils, which was later modified to be applicable to the PSC. Its core concept is to equate a PSC to a current disk with an average radius [32,33], and the basic form is

(9) $$L=\frac{\mu_{0}N^{2}D_{avg}d_{1}}{2}(\ln\frac{d_{2}}{\rho }+d_{3}\rho^{2})$$

where *d*_1_ = 1, *d*_2_ = 2.46, *d*_3_ = 0.2, *D_avg_* is the average diameter of PSC, and *ρ* is the filling factor:

(10) $$\begin{array}{l} D_{avg}=R_{in}+R_{o}=2R_{in}+Nw+(n-1)d\\ \rho =\frac{R_{o}-R_{in}}{R_{o}+R_{in}} \end{array}$$

The total length *l* of the PSC is regarded as the sum of the circumferences of *N* turns of the coil:

(11) $$l=N\pi D_{avg}=N\pi [2R_{in}+Nw+(n-1)d]$$

It is evident that the resistance of the PSC is

(12) $$R=\frac{\rho_{res}\pi N[2R_{in}+Nw+(n-1)d]}{wh}$$

where *ρ_res_* is the resistivity of the PSC material, the thickness of the PSC is *h*.

Since the EEC is driven with an alternating voltage signal *v*_EEC_(*t*) = *V*_EEC_sin(*ωt* + θ), the current *i*(*t*)_EEC_ in the EEC is written as

(13) $$i_{EEC}(t)=\frac{V_{EEC}}{\sqrt{R^{2}+{\left(\omega L\right)}^{2}}}\sin\left[\omega t+\theta -\arctan(\frac{\omega L}{R})\right]$$

Therefore, the induced voltage *V*_IEC_(*t*) in the IRC is formulated as

(14) $$v_{IRC}(t)=-M_{1}\frac{di_{EEC}(t)}{dt}=-\frac{V_{EEC}M_{1}\omega }{\sqrt{R^{2}+{\left(\omega L\right)}^{2}}}\cos\left[\omega t+\theta -\arctan(\frac{\omega L}{R})\right]$$

Since the EEC and the IEC are connected in series and have the same impedance, the expression of the current in the IEC is written as

(15) $$i_{IEC}(t)=\frac{v_{IRC}(t)}{2\sqrt{R^{2}+{\left(\omega L\right)}^{2}}}$$

Based on Equation (8), the mutual inductance between the IEC and the ERC is expressed as

(16) $$M_{2}=\sum_{i=1}^{N}\sum_{j=1}^{N}M\left[Rt_{i},Rr_{j},\left(z_{0}+H_{0}-\frac{\sigma H_{0}}{E}\right)\right]$$

where *H*_0_ represents the initial thickness of the elastic layer, *E* is the Young’s modulus of the elastic layer, and *σ* is the stress applied to the elastic layer.

The expression for the induced voltage *v*_ERC_(*t*) generated in the ERC is derived from Equation (14) and written as

(17) $$v_{ERC}(t)=-M_{2}\frac{di_{IEC}(t)}{dt}=-\frac{V_{IEC}M_{2}\omega }{\sqrt{R^{2}+{\left(\omega L\right)}^{2}}}\cos\left[\omega t+\theta -\arctan(\frac{\omega L}{R})\right]$$

From Equations (13), (14), (16) and (17), we come across an expression for the effective value *V*_E-ERC_ of the induced voltage generated by the ERC:

(18) $$V_{E-ERC}=\frac{M_{1}M_{2}\omega^{2}V_{EEC}}{2\sqrt{2}\left(R^{2}+\omega^{2}L^{2}\right)}$$

In this paper, the case and insulation are treated as rigid bodies. Consequently, *z*_0_ in Equation (8) is an invariant constant, and only *M*_2_ in Equation (17) varies with the stress. The signals output from the EWFS under different interfacial stresses can be calculated using Equation (17), thereby enabling the analysis of the EWFS’s response characteristics to interfacial stress.

### 3.2. Simulations and Analysis

A series of simulations have been conducted based on the formulated expressions in an effort to investigate the EWFS response to interfacial stresses under different frequencies of *v*_EEC_(*t*). By analyzing Equation (12) and (18), *V*_E-ERC_ is a monotonically increasing function of *h*, indicating a positive correlation between the sensitivity of the EWFS to interfacial stress and *h*. Typically, the thickness specifications of FPCB copper foil include 0.0175 mm, 0.035 mm, 0.07 mm and 0.105 mm, etc. Considering the sensitivity of the EWFS and the flexibility of the FPCB, the PSC in the FPCB adopts a copper foil thickness of 0.035 mm. For the copper foil with a thickness of 0.035 mm, the typical line width is 0.1 mm and the line spacing is 0.1 mm. The simulation parameters are tabulated in Table 1.

**Figure 4:**
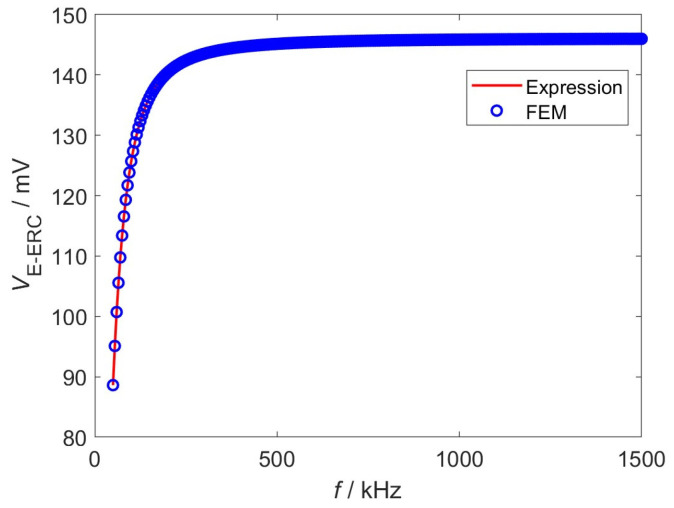
EWFS signals.

In a bid to verify and evaluate the established expression of the EWFS, the Normalized Root Mean Squared Deviations (NRMSD) between the computed results and the finite element method (FEM) results is calculated, whilst the computing time is obtained to evaluate the computation efficiency [34]. The expression results and the FEM results are shown in Figure 4.

It can be seen from Figure 4 that the computed expression signals have good agreement with the results from the FEM. The NRMSD of the expression is calculated to be 1.32%. The expression calculation takes 35.3 s, whereas the FEM costs considerably more time (1396.8 s). As a result, the expression is preferred for the following theoretical investigation.

**Figure 5:**
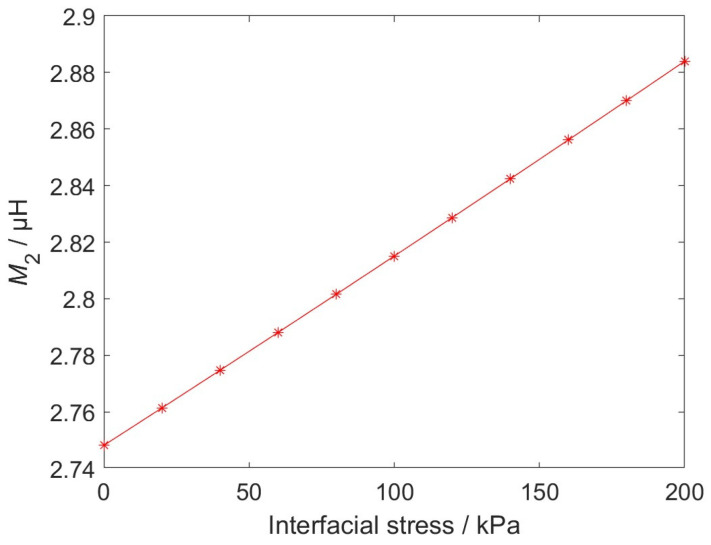
Interfacial stress vs. *M*_2_.

As the only parameter varying with the interfacial stress, the relationship between *M*_2_ and interfacial stress is shown in Figure 5. It can be observed from Figure 5 when the interfacial stress increases from 0 kPa to 200 kPa, *M*_2_ increases from 2.748 μH to 2.884 μH. *M*_2_ exhibits an extremely strong linear positive correlation with interfacial stress, indicating that the EWFS output signal responds to interfacial stress in a linear and stable manner.

**Figure 6:**
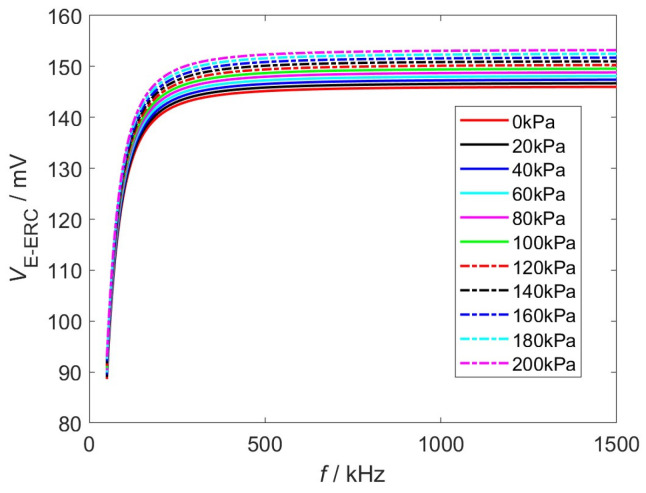
*V*_E-ERC_ vs. the interfacial stress against the excitation frequency.

In order to investigate the response characteristics of interface stress to signals of different frequencies, the EWFS output signals are calculated in the range of 50 kHz to 1500 kHz with a step size of 1 kHz. The raw signals are shown in Figure 6. *V*_E-ERC_ under all stress conditions exhibits a trend of rapid growth followed by saturation with increasing frequency: starting from 50 kHz, *V*_E-ERC_ rises sharply with the increase in frequency; after the frequency exceeds 300 kHz, *V*_E-ERC_ gradually levels off and enters a steady-state plateau region, with a significantly reduced variation amplitude of *V*_E-ERC_.

**Figure 7:**
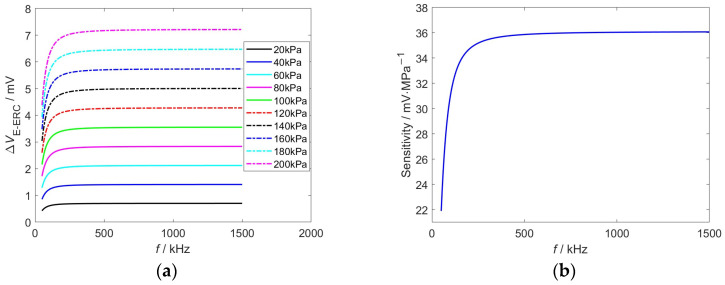
The differential signal and sensitivity of EWFS: (**a**) differential signal; (**b**) sensitivity.

To further enhance the stress-induced signal variation, the differential signal is obtained by subtracting the stress-free signal from the signal corresponding to interfacial stress, as shown in Figure 7a. Similar to *V*_E-ERC_, Δ*V*_E-ERC_ undergoes a rapid rise at low frequencies (<300 kHz) followed by gradual saturation at high frequencies. Interestingly, the steady-state amplitude of Δ*V*_E-ERC_ has a monotonic increase with interfacial stress reflecting the sensitive response of Δ*V*_E-ERC_ to interfacial stress. The trend of sensitivity varying with frequency is consistent with Δ*V*_E-ERC_, as shown in Figure 7b. When the frequency exceeds 300 kHz, the sensitivity reaches the maximum value of 36 mV·MPa^−1^.

## 4. Experiments and EWFS Performances

### 4.1. The EWFS System

**Figure 8:**
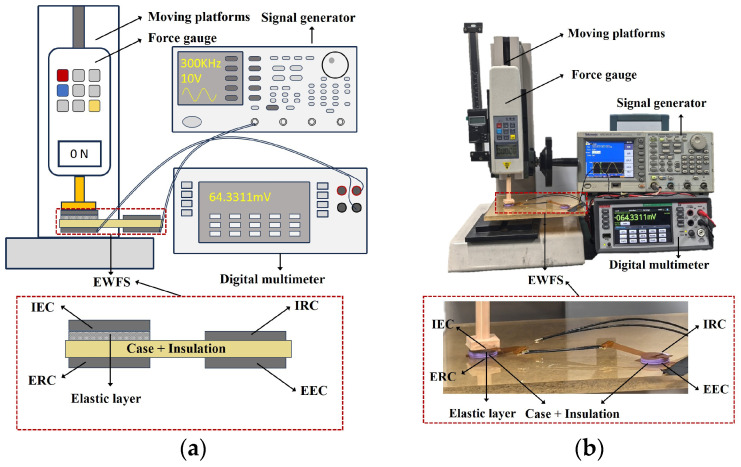
The EWFS system: (**a**) schematic illustration; (**b**) practical picture.

The EWFS system for interface stress monitoring consists of a signal generator (Tektronix AFG3022C, Beaverton, OR, USA), a vertical stress-testing machine with a force gauge (Handpi Instruments HP-1000N, Wenzhou, China), a fabricated EWFS and a digital multimeter (Keithley DMM6500, Solon, OH, USA). The schematic illustration and practical picture of the system setup are shown in Figure 8. The EWFS consists of four FPCBs and one polydimethylsiloxane (PDMS) elastic layer. PDMS is a frequently chosen material for the elastic layer in flexible and wearable electronic systems due to its unique combination of properties, which offer significant advantages over other elastomeric material. PDMS offers remarkable elasticity and stretchability, biocompatibility, excellent chemical inertness and stability, and tunable mechanical properties [35,36]. The parameters of the EWFS are the same as those listed in Table 1. In the experiment, acrylonitrile butadiene styrene (ABS) specimens with a thickness of 3 mm are used to simulate the case and the insulation. In combination with the simulation conclusions and equipment parameters, the frequency of *v*_EEC_(*t*) is set as 300 kHz. It is noteworthy that, in the experiment, the metal base of the pressure machine must maintain a certain distance from the EWFS to avoid electromagnetic interference caused by direct contact.

### 4.2. Linearity and Sensitivity of EWFS

**Figure 9:**
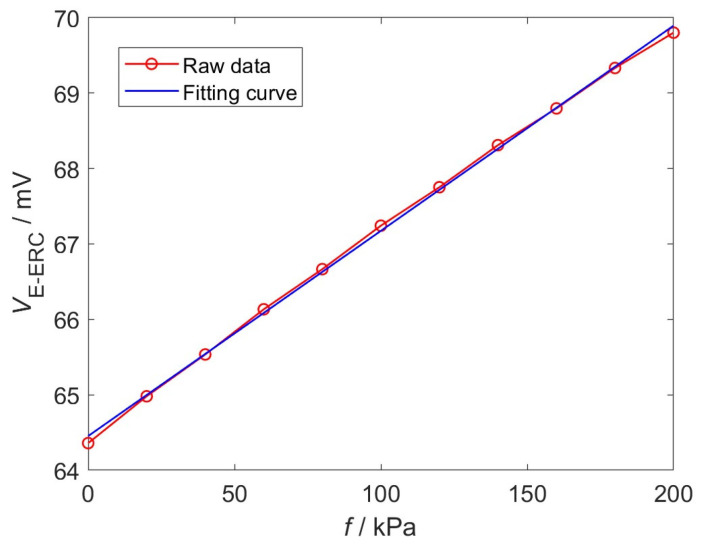
Raw data and fitting curve.

The EWFS signals corresponding to interface stresses from 0 kPa to 200 kPa are collected in increments of 20 kPa. The results are shown in Figure 9. The amplitude of *V*_E-ERC_ is smaller than the amplitude of the simulated result in Figure 7, primarily due to the voltage division across the connecting cables between the signal generator and the EEC, and between the IRC and the IEC in the circuit. Comparable to the simulation results, a good linear relationship is observed between the EWFS signals and the interfacial stress. Linear fitting is performed on the original signals, and the linearity error is calculated using the following expression [19]:

(19) $$\delta_{L}=\frac{\Delta L_{\max}}{Y_{FS}}\times 100\%$$

where *Y_FS_* represents the full-scale output of the EWFS and Δ*L*_max_ is the maximum deviation value between the raw data and the fitting curve. The fitting curve of the raw data is expressed as

(20) y=27.2x+64.453

From Equations (19) and (20), the sensitivity of the EWFS is determined to be 27.2 mV·MPa^−1^, and the linearity error is 1.73%, indicating that the EWFS exhibits excellent linearity.

### 4.3. Hysteresis Error of EWFS

Testing the hysteresis error of the EWFS is essential. The hysteresis error is one of the key static performance indicators of the EWFS, which reflects the inconsistency between its output responses under the interface stress during the loading and unloading processes. For long-term monitoring of interfacial stress, excessive hysteresis error will lead to obvious deviations between measured values and real interface stress states, and may even cause misjudgment of structural health conditions and potential safety risks. Therefore, evaluating the hysteresis error is necessary to ensure the measurement accuracy and reliability of the EWFS.

**Figure 10:**
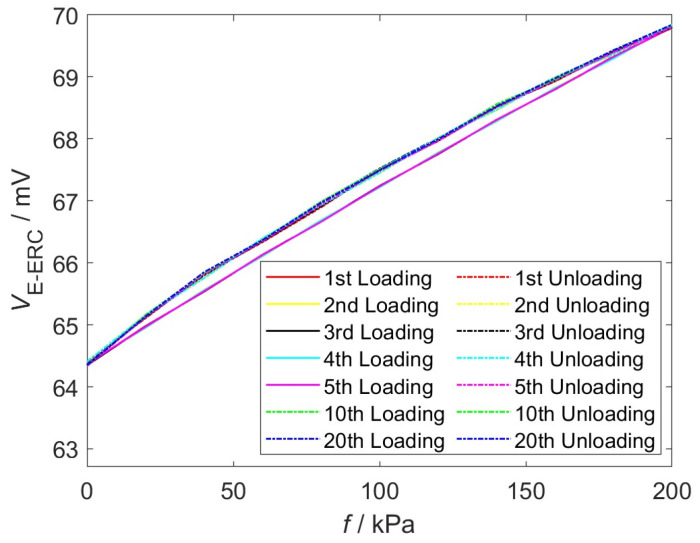
Twenty loading–unloading tests.

Typically, the evaluation of hysteresis error is completed by comparing the differences in EWFS output signals during the loading and unloading processes. The smaller the hysteresis error, the more stable the EWFS, and the more accurate its response to interfacial stress during loading and unloading processes. The hysteresis error is calculated with the expression [19]

(21) $$\delta_{H}=\frac{\Delta H_{\max}}{Y_{FS}}\times 100\%$$

where *Y_FS_* represents the full-scale output of the EWFS and Δ*H*_max_ is the maximum output difference between the loading and unloading processes. In order to quantitatively assess the impact of hysteresis error, the sensor element is subjected to 20 identical tests, illustrated in Figure 10.

It can be calculated from Equation (21) that *δ_H_* of the 1st loading–unloading, 10th loading–unloading, 20th loading–unloading, average value, and maximum value are 2.54%, 2.62%, 2.57%, 2.61%, and 2.67, respectively, indicating good hysteresis characteristics. The relatively low hysteresis error of the EWFS is due to the synergistic effect of the elastic PDMS and FPCB under practical cyclic loading conditions. Hysteresis in materials, fundamentally, represents the energy dissipated as heat during a loading–unloading cycle, reflecting irreversible changes within the material’s microstructure. Rapid stress relaxation in PDMS, characterized by its ability to quickly return to its original state after deformation, minimizes internal friction and thus energy dissipation during loading and unloading cycles. Under loading conditions, the FPCB exhibits low viscoelastic dissipation, meaning it undergoes minimal internal energy loss. Furthermore, the *V*_E-ERC_ of the EWFS during the 20 repeated loading–unloading experiments exhibits highly overlapping curves, demonstrating a high degree of output consistency. Therefore, in the complex and varying internal stress environment of the SRM, the EWFS can accurately measure interface stress.

### 4.4. Static Stability of EWFS

**Figure 11:**
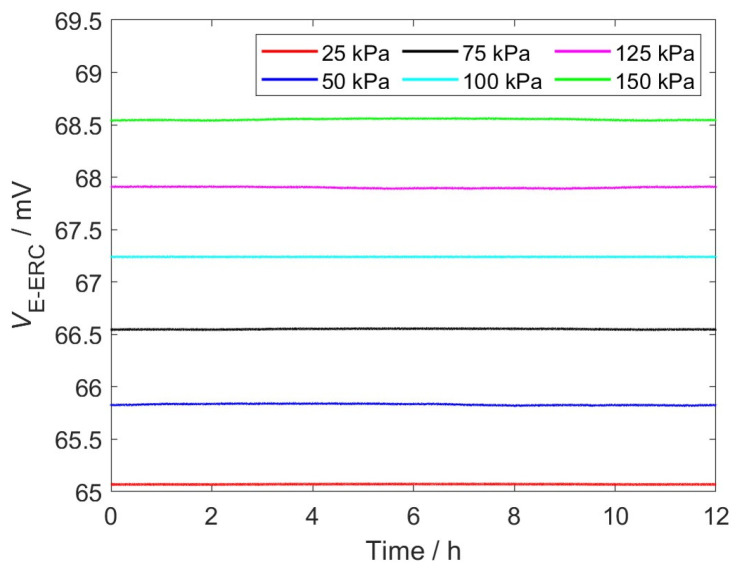
The signal output by the EWFS under 25 kPa, 50 kPa, 75 kPa, 100 kPa, 125 kPa and 150 kPa stress conditions within 12 h.

Interface stress sensors are generally difficult to be calibrated after installation. During long-term continuous monitoring, even small drifts in the sensor output may be misinterpreted as variations in structural stress, thereby leading to erroneous assessments of the SRM interface stress. To ensure measurement reliability, sensors are required to exhibit excellent static stability. In the experiment, constant stresses of 25 kPa, 50 kPa, 75 kPa, 100 kPa, 125 kPa and 150 kPa are separately applied to the EWFS, and the EWFS output signals are continuously recorded within 12 h using the digital multimeter. The results are presented in Figure 11.

As can intuitively be observed from Figure 11, during the 12-h monitoring period, the response signals under all the interface stress conditions exhibit high stability, with only minor random fluctuations. To quantify the stability of the EWFS, the Relative Standard Deviation (RSD) is employed, which is written as follows [19]:

(22) $$\mathrm{RSD}=\frac{\sqrt{\frac{\sum_{i=1}^{n}{\left(V_{i}-V_{avg}\right)}^{2}}{n-1}}}{V_{avg}}\times 100\%$$

where *n* represents the number of output voltage data points collected within 12 h and *V_i_* and *V_avg_* are the measured and average output voltage under the same stress, respectively. It can be derived from Equation (22) that RSD under the interfacial stresses of 25 kPa, 50 kPa, 75 kPa, 100 kPa, 125 kPa and 150 kPa are 0.015%, 0.023%, 0.018%, 0.029% and 0.022%, respectively. This indicates that the EWFS has the excellent stability under different interface stress for the long-term health monitoring of SRM.

## 5. Conclusions

In this paper, a novel EWFS for in situ monitoring of the interface stress without damaging the case of SRM has been proposed. The basic principle of EWFS for interface stress monitoring is elaborated. The expression of the EWFS output signal for interface stress is derived, and verified through FEM. It is found that the expression has the advantage of high computational efficiency. From the calculation results, it is inferred that the signal is proportional to the interface stress. Subsequently, the EWFS is fabricated and the experimental system is set up. By applying interface stress to the case and insulation, experimental data of the EWFS under different stress conditions are obtained. The results imply that the EWFS composed of FPCB and PDMS achieves a sensitivity of 27.2 mV·MPa^−1^, a linearity error of 1.73%, a maximum hysteresis error of 2.67%, and a stability error of 0.029% within 12 h. The results from simulations and experiments indicate the feasibility and applicability of the proposed EWFS for monitoring the interface stress in the SRM. Although the EWFS exhibits excellent stability in the 12-h experiment, it does not indicate that the EWFS still maintains outstanding stability under long-term service conditions. Therefore, the EWFS structure will be further optimized to improve its sensitivity. Meanwhile, the drift characteristics of the EWFS during long-term service and the influence of material degradation on output signals will be investigated, so as to promote the practical application of the EWFS in long-term stored SRM.

## Figures and Tables

**Table 1:** The simulation parameters.

*R_in_*	*w*	*d*	*N*	*z*_0_	*H*_0_	*V*_EEC_	*E*	*h*	*ρ_res_*
0.4 mm	0.1 mm	0.1 mm	41	3 mm	2 mm	10 V	2 MPa	0.035 mm	1.75 × 10^−8^ Ω·m

## Data Availability

The data presented in this study are available on request from the corresponding author after obtaining permission of an authorized person.
